# Survey on Heavy Metals Contamination and Health Risk Assessment in Commercially Valuable Asian Swamp Eel, *Monopterus albus* from Kelantan, Malaysia

**DOI:** 10.1038/s41598-019-42753-2

**Published:** 2019-04-23

**Authors:** Ai Yin Sow, Ahmad Ismail, Syaizwan Zahmir Zulkifli, Mohammad Noor Amal, Kamarul Ariffin Hambali

**Affiliations:** 1 Faculty of Agro-Based Industry, Universiti Malaysia Kelantan, Jeli Campus, Locked Bag No. 100, 17600 Jeli, Kelantan Malaysia; 2 Biology Department, Faculty of Science, Universiti Putra Malaysia, 43400 UPM Serdang, Selangor Malaysia; 3 Faculty of Earth Science, Universiti Malaysia Kelantan, Jeli Campus, Locked Bag No. 100, 17600 Jeli, Kelantan Malaysia

**Keywords:** Freshwater ecology, Freshwater ecology, Environmental monitoring, Environmental monitoring, Environmental impact

## Abstract

This work investigates the metals concentration in the tissues of Asian swamp eel, *Monopterus albus*. Five selected tissues, including liver, gill, bone, skin, and muscle were examined for the concentration of Zn, Cu, Cd, Pb, and Ni. The concentrations of Cd and Pb were found high in the muscle tissues of the eels. Additionally, high amounts of Zn and Cu metals were observed in the liver, whereas the Cd, Pb, and Ni metals were highly detected in gill. The accumulation of Zn, Cu, Cd, Pb, and Ni in both skin and bone of the eel seems to vary between seasons. Low levels of Zn, Cu, and Ni were identified in the muscle tissues of the eels. This study revealed that the concentration of Cd and Pb in the muscle tissues of Asian swamp eels exceeded the permissible limits by the US EPA, suggesting the consumption of the muscle may be hazardous and can severely affect one’s health.

## Introduction

Fish is widely consumed by the general population because it is one of the major food sources found worldwide. Moreover, its wide consumption owes to the fact that it is also one of the cheapest sources of protein considering that protein is highly essential for the human body. In this study, Asian swamp eel, *Monopterus albus* or also known as ‘paddy eel’ by the locals, had been chosen as the subject, considering that it is widely consumed amidst the Kelantanese community. Asian swamp eel is a freshwater fish, which belonging to the Synbranchidae family, resulting in different between the anguillid species (eel family), which is commonly found in marine environments. Asian swamp eel is very popular in Kelantan due to its pleasant taste, soft muscle composition, and well-known medicinal benefits. The consumption of fish has many beneficial because it is a source that provides high quality of protein, which includes two types of omega 3 poly-unsaturated fatty acids, namely eicosapentaenoic acid (EPA) and docosahexaenoic acid (DHA)^1,2^. In fact, it was discovered that this fish has substantial medicinal benefits that can be very helpful in fighting several diseases, such as rheumatoid arthritis^3,4^. The market values for paddy eel ranged from USD 4.88-USD 6.10, whereas for anguillid species, price ranges from USD 3–15/kg, which ranks the eel among the most precious species of food fish.

A recent study reported that poor management of garbage disposal, human activity, and manufactured waste, which appear to be the cause of pollution, pose detrimental effects to the environment. Besides, one cannot deny that paddying, an agricultural activity has contributed to a high number of hazardous waste. In this case, the existence of pesticides, such as apple snail (*Pomacea* sp), is regarded to be very damaging to the harvest^5^. This deleterious scenario may worsen given that certain environmental conditions can increase the tendency for heavy metals to seep into water and paddy soil, which then produce more concentrated toxic elements and non-biodegradable damages^6^. The Asian swamp eels have been severely affected when heavy metals and metalloids are present in higher concentrations. Rajeshkumar & Li^7^. stressed that the gill and liver are outstanding target organs because the gill reflects the concentration of metals in water, while the liver represents the storage of metals. Hence, frequent consumers of Asian swamp eel are exposed to potential health hazard due to toxic accumulated in the eel^8^. For instance, it was discovered that the efficient usage of heavy metals from infected water and food may vary according to the aspects of ecological demands, as well as metabolism and pollutant gradients of water, food, and sediment^9^. Apart from that, other factors need to be considered as well, inclusive of salinity, temperature, and other environmental parameters.

Heavy metals have been widely used for centuries by humans for various purposes. The metals in the system of human body can be divided on the basis of two aspects, which are: essential and non-essential metals. Essential metals include Fe, Cu and Zn are crucial for the development of living systems. On the other hand, non-essential metals, such as Pb and Cd, are known to be toxic when consumed excessive^10^. Since fish is a main source of food consumed by human beings^4^, it is expected that a high amount of heavy metals is likely to accumulate in their body tissues. This continuous exposure to heavy metals is believed to inflict the human body with acute diseases, where the assimilation of heavy metals is one of the causes of cancer^11^. For instance, the high concentrations of Zn may cause health problems such as skin irritations, vomiting, and stomach cramps^12,13^, while, too much of Ni could lead to cancer of lungs and kidney^12,14^.

Thus, this study selected Asian swamp eel as the bio-indicator agent in paddy cultivation areas. Moreover, the Kelantanese community had been chosen as the subject of this study because it is a known fact that this community frequently consume this eel, when compared to other types of marine life. However, it can be assumed that they are unaware of the varied levels of heavy metals present in different types of fishes, considering the scant information made available to the public pertaining to the contaminant levels in commercial fish. Hence, this study analysed the level of heavy metals from several parts of Asian swamp eel tissues, including the gill, muscle, skin, liver, and bone. With that, this case study, apart from determining the concentration of heavy metals in paddy eel, looked into the types of heavy metals found in different paddy seasons.Therefore, the main purpose of this study was to^1^ investigate heavy metals concentrations in tissues of Asian swamp eel and^2^ to undertake a health risk assessment of the consumption of heavy metals via Asian swamp eel. Moreover, the muscle of the eel was chosen to determine the potential harmful effects following its consumption to human beings.

## Results

### Accumulation of heavy metals in selected tissues of asian swamp eel

**Figure 1:**
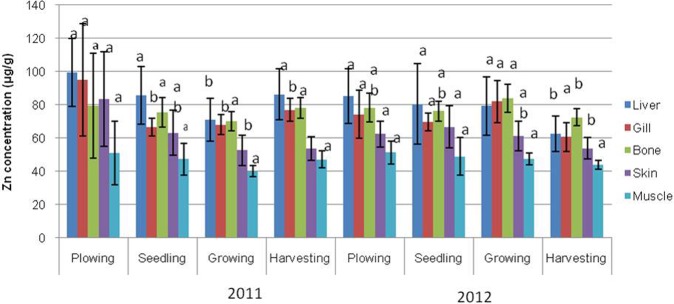
Mean concentrations (µg metal/g dw) and standard deviations of Zn in the tissues of Asian swamp eels for the year 2011 and 2012. Note: *Post-Hoc*: Mean metal concentrations of different parts of tissues sharing a common letter for a particular metal present no significant differences, p > 0.05.

**Figure 2:**
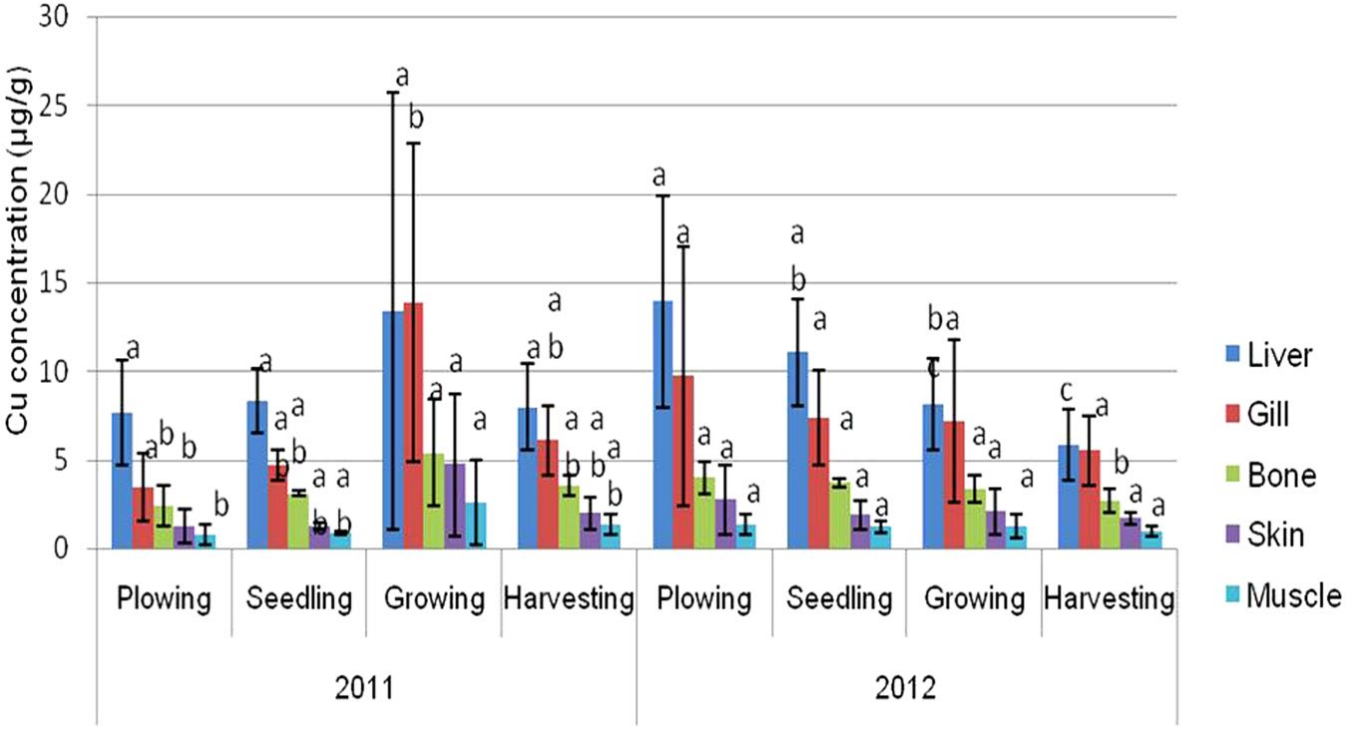
Mean concentrations (µg metal/g dw) and standard deviations of Cu in the tissues of Asian swamp eels for the year 2011 and 2012. Note: *Post-Hoc*: Mean metal concentrations of different parts of tissues sharing a common letter for a particular metal present no significant differences, p > 0.05.

**Figure 3:**
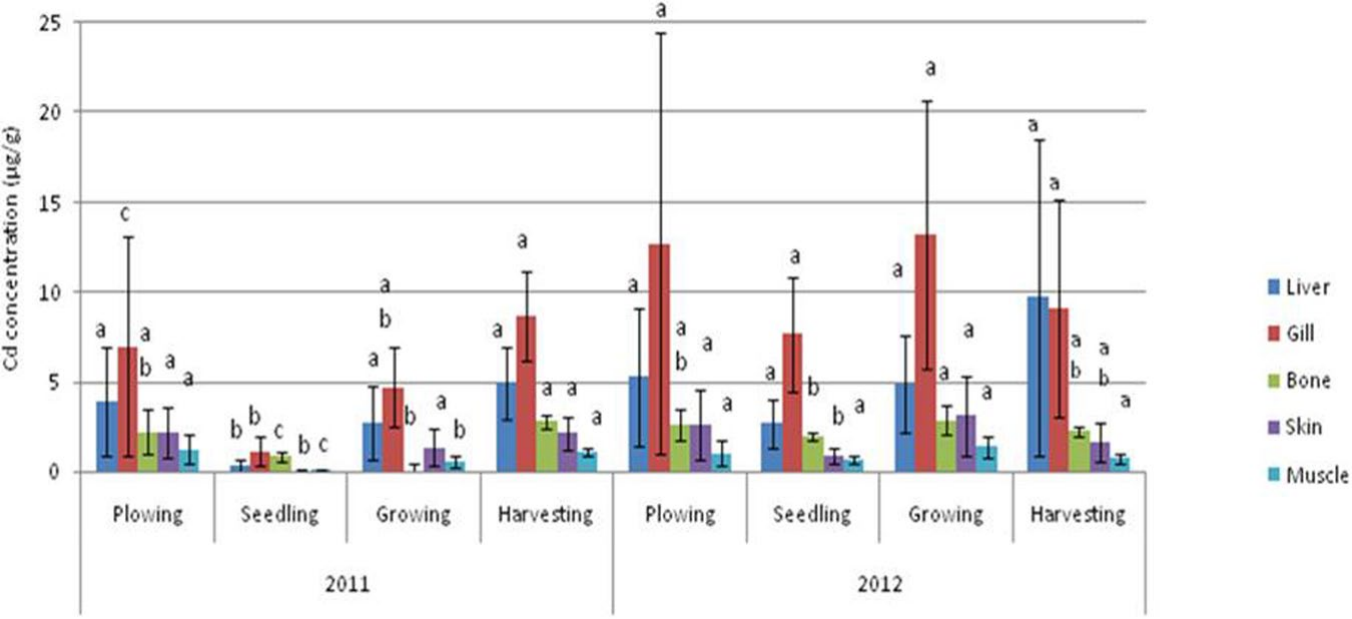
Mean concentrations (µg metal/g dw) and standard deviations of Cd in the tissues of Asian swamp eels for the year 2011 and 2012. Note: *Post-Hoc*: Mean metal concentrations of different parts of tissues sharing a common letter for a particular metal present no significant differences, p > 0.05.

**Figure 4:**
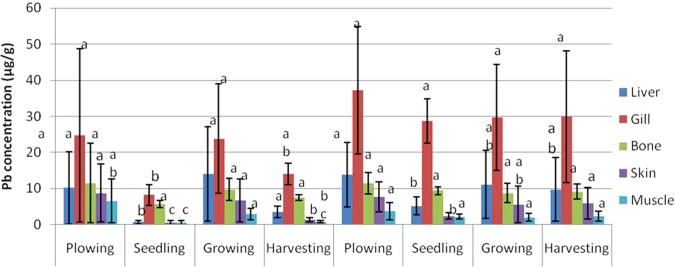
Mean concentrations (µg metal/g dw) and standard deviations of Pb in the tissues of Asian swamp eels for the year 2011 and 2012. Note: *Post-Hoc*: Mean metal concentrations of different parts of tissues sharing a common letter for a particular metal present no significant differences, p > 0.05.

**Figure 5:**
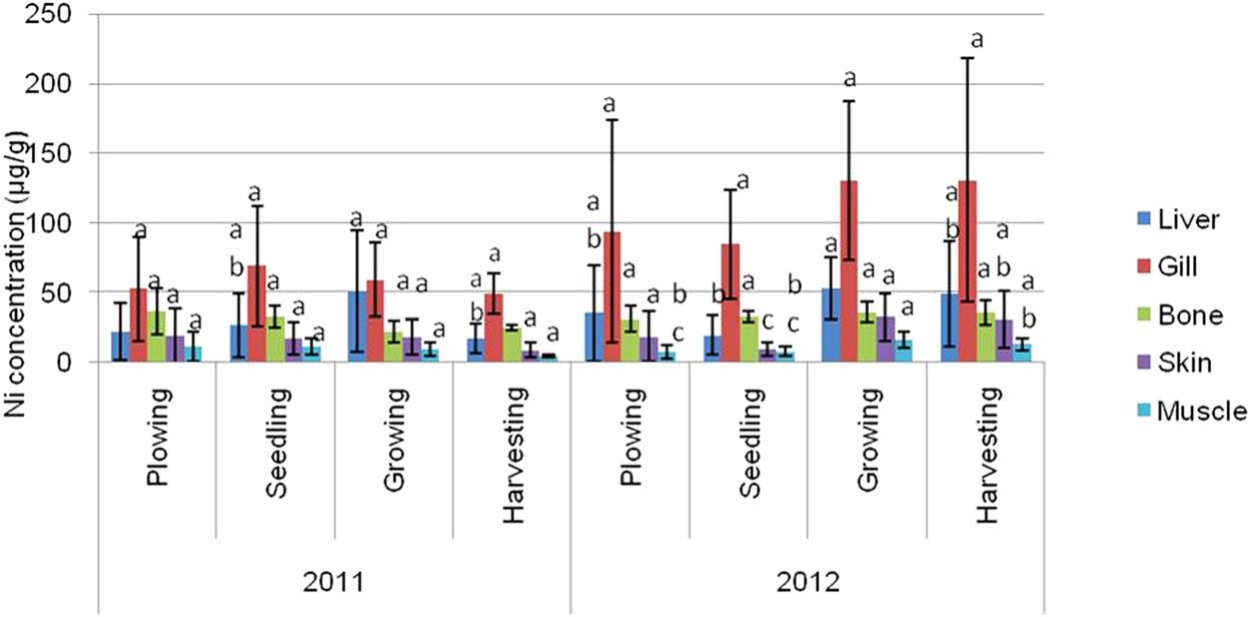
Mean concentrations (µg metal/g dw) and standard deviations of Ni in the tissues of Asian swamp eels for the year 2011 and 2012. Note: *Post-Hoc*: Mean metal concentrations of different parts of tissues sharing a common letter for a particular metal present no significant differences, p > 0.05.

The mean values of metal concentrations (Zn, Cu, Cd, Pb, and Ni) for the varying parts of Asian swamp eel tissues, such as liver, gill, bone, skin, and muscle are presented in Figs 1–5. The highest mean values of Zn concentration during the ploughing season in the liver of the eels were 99.58 µg/g dw and 85.36 µg/g dw for years 2011 and 2012. Further analysis performed on all the metals showed that the liver tissues contained the highest Cu metal for both years. On top of that, the topmost mean values of Cd metal for four different paddy seasons found in the gill tissues were 5.36 µg/g dw for 2011, and 10.67 µg/g dw for 2012. Meanwhile, similar finding for Pb metal was observed in the gill tissues, which displayed the highest mean values for all four paddy seasons in both years, with 75.57 µg/g dw for 2011, and 109.26 µg/g dw for 2012. As for Ni, the highest concentration was found in gill tissues during the ploughing season, with 24.70 µg/g dw for 2011, and 37.27 µg/g dw for 2012.

One significant outcome that emerged from the analyses is that the highest metal concentration of Zn was discovered in the bone of the eels was recorded at 79.60 µg/g dw for the ploughing season in 2011, while 84.01 µg/g dw for the growing season in 2012. Nevertheless, no significant (p > 0.05) variance was noted among all the four seasons in years 2011 and 2012. Further, Cd metal appeared to have the lowest concentration, when compared to other metals found in the bone of the eels, which had been recorded at 0.84 µg/g dw and 1.98 µg/g dw for seedling seasons in 2011 and 2012, respectively. Meanwhile, the findings for skin of the eels had been similar to those of the bone, which displayed the highest accumulation of Zn metal during the ploughing season at 83.61 µg/g dw and the seedling season at 66.88 µg/g dw for both years. Nonetheless, the concentration of Cd metals was low in skin tissues during the seedling season particularly with records of 0.076 µg/g dw and 0.920 µg/g dw for 2011 and 2012, respectively. On top of that, the muscle exhibited the lowest concentration of metals at every paddy season. Therefore, the single most striking observation describes that most metals were accumulated in high percentages during the ploughing and growing seasons.

As presented in Fig. 1, no significant difference (p > 0.05) was noted in the metal concentration of Zn in the study of different parts of tissues (liver, gill, bone, skin, and muscle) for all four paddy seasons in the years 2011 and 2012. In addition, none of the variance for Cu metal had been statistically significant (p > 0.05) in the tissues of the eels for all of the four paddy seasons in the year 2011. Contrary, Cu metal in the year 2012 displayed a significant difference (p < 0.05) in liver tissues among the paddy seasons (Fig. 2). However, some variations in Cd metal concentrations were observed in the tissues of the eels for all the paddy seasons in 2011 (Fig. 3). Moreover, the Pb metal in tissues (liver, gill, bone, skin, and muscle) for all the paddy seasons in the year 2011 did not reveal any significant difference (p > 0.05), but the Pb metal illustrated a great variance (p < 0.05) in skin and muscle of the eels for the paddy season in year 2012 (Fig. 4). On top of that, a significant difference (p < 0.05) was observed in Ni metal concentrations in the skin and muscle of the eels for all paddy seasons in 2011 (Fig. 5). Nevertheless, no significant variance (p > 0.05) was detected for Ni metal concentrations in the tissues of the eels for all paddy seasons in 2012.

The estimated daily intake values for 2011 and 2012 are outlined in Table 1, where the average daily intake values in 2011 for Zn: 25.37–32.34 µ/kg/day, for Cu: 0.490–1.640 µ/kg/day, for Cd: 0.053–0.800 µ/kg/day, for Pb: 2.993–7.363 µ/kg/day, and for Ni: 0.404–4.170 µ/kg/day. The average estimated daily intake values in 2012 for Zn: 2.782–32.47 µ/kg/day, for Cu: 0.629–0.870 µ/kg/day, for Cd: 0.446–0.887 µ/kg/day, for Pb: 4.970–10.310 µ/kg/day and for Ni: 1.282–2.330 µ/kg/day. In comparison with the standard guidelines (Table 1), the estimated daily intakes of each metal in Asian swamp eel showed below the standard range except Pb metal in Asian swamp eel collected on harvesting season in 2011.

**Table 1:** Daily intake of heavy metals (µ/kg/day) in Asian swamp eel muscle for an individual of 50 kg in Malaysia.

Year	Season	N	Zn	Cu	Cd	Pb	Ni
2011	Ploughing	38	32.34	0.490	0.800	7.320	4.170
	Seedling	9	29.94	0.569	0.053	7.363	0.404
	Growing	24	25.37	1.640	0.370	6.230	1.890
	Harvesting	11	29.92	0.876	0.721	2.993	0.523
2012	Ploughing	32	32.47	0.870	0.650	4.970	2.330
	Seedling	8	31.04	0.766	0.446	5.167	1.397
	Growing	21	30.07	0.769	0.887	10.31	1.282
	Harvesting	10	27.82	0.629	0.471	8.248	1.501
	Reference Doses (RfDo) in µ/kg/day unit		300^a^	40^a^	1^a^	3.57^b^	20^c^

Remark: a^46^, b^47^, c^48^.

## Discussion

### Bioaccumulation of Zn, Cu, Cd, Pb, and Ni in the tissues of asian swamp eel

This study conducted for a two-year observation period based on each paddy cycle, in order to assess the concentrations of Zn, Cu, Cd, Pb, and Ni accumulation in the selected tissues (muscle, skin, bone, gill, and liver) of Asian swamp eels. The observation made in the four seasons of paddy cycle, which are ploughing, seedling, growing, and harvesting.

Zn is an essential element for metabolism activity in organisms^15^. Zn can be transmitted in the aquatic food chain, which in the end is believed to produce deleterious effect to the human health. Maret^16^ recognized Zn as an essential element that promotes a wide variety of enzymes and other cell components, aside from ascertaining the vital functions of living organisms. In this study, Zn concentrations were found to be the highest in liver tissues of Asian swamp eel for all four paddy seasons in both the years of 2011 and 2012. This is not surprising considering that the liver and gills are classified as target organs that are expected to have substantially high levels of heavy metals^16^. This possibly exerts hazardous effects to the eels, especially during its egg and juvenile stages by reducing the survival of eel embryos and deformed or developed embryos died during embryonic development^17^. Moreover, the dietary habits of this eel may also have a role in increasing the level of Zn concentration in liver tissues. Asian swamp eels mostly feed on small fishes, invertebrates, apple snails, and other organisms as dietary food. According to Long *et al*.^18^, a strong correlation was found in Zn concentrations between zooplankton and fish. In a comprehensive study of the similar topic, it was recorded the accumulation of Zn metal in the liver of bream stream (*Abramis brama*) tissues was 78.40 mg/kg dw and 82.50 mg/kg dw, respectively^19^. These results contradict the findings of the present study because the concentrations recorded in prior studies are lower than those of the present study. This might due to the application of agrochemical fertilizers during different paddy seasons in both years, subsequently, increase the bioaccumulation of Zn in tissues of Asian swamp eel. Moreover, it is widely known that liver has an important function in the processes of accumulation, uptake, and storage of heavy metals. Metallothioneins comes from the family of low-molecular weight, whereby cysteine-rich proteins are prone to have high affinity towards divalent cations^20^. In addition, huge amounts of metallothionein proteins induction may take place in the fish liver^21^. Most often than not, the adoption of agrochemical fertilizers and pesticides during the paddy seasons (ploughing, seedling, growing, and harvesting) may increase the amount of Zn metal in paddy soils. On another note, it is crucial to understand that Asian swamp eel exposed at the paddy fields for a longer duration of time tend to produce higher Zn level in the liver tissues. According to Kemubu Agricultural Development Authority^22^, Zn is an element that has been commonly utilized to produce agrochemical fertilizers. In years 2011 and 2012, muscle tissues were found to accumulate low Zn concentrations for all the paddy seasons, which ranged between 40.15 and 51.38 µg/g dw. Next, a research conducted by Kalay *et al*.^23^ discovered that the Zn level was ranged from 16.1–25.8 µg/g dw for red mullet (*Mullus barbatus*), 18.0–33.60 µg/g dw for blue runner (*Caranx crysos*), and 23.50–30. 90 µg/g dw for flathead grey mullet (*Mugil cephalus*) in a study from Northeast Mediterranean Sea. Meanwhile, the Zn concentration in muscle tissues for the present study was found to be higher compared to the outcome reported in the study conducted by Kalay *et al*.^23^. Moreover, a high concentration of Zn was found in muscle tissues of goldfish (*Carassius auratus*), which was recorded at 130 mg/kg dw^24^. Arai *et al*.^25^ reported high levels of Zn was found in the Indonesian shortfin eel (*Anguilla bicolor bicolor*) from Bukit Merah, Perak and Penang River, Penang, Malaysia, which was recorded at 65.9 ± 10.6 µg/g and 75.3 ± 18.9 µg/g, respectively. The different concentrations of Zn in muscle tissues may be caused by several factors, such as locality (natural or anthropogenic sources), the physiological conditions of the fish (age and size), and seasonal variations^26^. On top of that, Malaysian Food Regulation has set the permissible Zn limit at 100 µg/g, which suggests that muscle tissues are not harmful for consumption^27^.

The highest Cu metal concentrations were reported in the liver of Asian swamp eel for all the paddy seasons in both years of 2011 and 2012. Similar results were found for Zn metal. In relation to this, a preliminary study on heavy metals in Asian swamp eels managed to detect the highest concentration of Cu metal in the liver^5^. Cu plays a vital role in the metabolism of living organisms. Hence, compound fertilizers that consist of Cu and Zn as part of their elements may have increased the Cu level in the paddy eel^5^. It was further reported that the essential metals, which are Cu, Zn, and Fe, can get through via three pathways, namely, food, water, and sediment^28^. However, the effectiveness uptake of heavy metals might differ due to several factors, including ecological needs and metabolism of animals; contaminant gradients of water, food, and sediment; as well as environmental guidelines, such as pH, salinity, temperature, and interacting agents^29^. In this study, the ecological requirements have been considered as a crucial root of heavy metals accumulation in the liver. Moreover, Asian swamp eel has the ability to burrow into a depth of 1.5 m in paddy soils, thus requiring them to stay in the hole of paddy soil during the day and to come out at night to search for food. Hence, this indicates that the exposure period of this eel to the contaminants is longer at night. Meanwhile, the Cu level in the muscle ranged from 0.78 to 2.60 µg/g dw for both years of 2011 and 2012. The values are within the permissible limit of Cu level in fish, which established at 30 µg/g by the Malaysian Food Regulation^30^. Nevertheless, no correlation was displayed between metal concentrations in water and fish muscle^30^. Moreover, muscle is a poor indicator to determine the minimum level of Cu and Zn contamination^31^.

Cd is a non-fundamental and severely hazardous metal that can produce negative effects on eel physiology, especially on its reproduction^32^. Generally, Cd is widely known as an industrial and green pollutant that causes unnecessary effects to certain organs in humans^33^. On top of that, gills are also described as the target organ that can accumulate high concentration of heavy metals from its surroundings. Gill tissues appeared to produce the highest Cd metal concentration for all paddy seasons in the two years’ duration. In addition, that the concentration of metals in water is best represented by the gills^34^. However, gill refers to an uptake site of waterborne ions, where metal concentrations tend to increase particularly at the beginning of exposure and before the metal gets into other parts of a particular organism^35^. Furthermore, the presence of thick layer of mucus on the surfaces of gill and the complication in getting rid of the excess mucus may result in high concentration of heavy metals, especially Cd. Meanwhile, it was discovered that the increase of Cd level in fish tissues is the result of the increased accumulations of Zn in the tissues of tilapia (*Tilapia nilotica*)^36^. The large surface area of the gills facilitates adsorption of Cd and Pb onto the surface of the gills during respiration and osmoregulation processes^37^. In this case, muscle tissues were found to accumulate minimum Cd concentration for all the paddy seasons. According to Le *et al*.^26^, the Cd level in the muscle of giant mottled eel (*A. marmorata*), should be in the range of 0.02 to 0.13 µg/g dw, which indicates a lower concentration, as compared to the result of this present research (0.08 to 1.41 µg/g dw). Nevertheless, the Cd concentrations of this study are still somewhat above the maximum permitted concentration in fish flesh for human consumption in Malaysia (1.0 µg/g)^34^. These results further corroborate the idea that there is a potential for Cd pollution to occur in the areas of paddy cultivation in Kelantan. The intensive use of rock phosphate as fertilizers in paddy cultivations areas has gradually increased the Cd contents, which subsequently affects the eel population.

The four paddy seasons observed revealed high Pb and Ni concentrations in gill tissues of paddy eel. Tchounwou *et al*.^38^, described that Pb deposition in aquatic ecosystem is the result of superficial soil erosion and atmospheric deposition process. As mentioned previously, both these metals are non-fundamental; hence, only low amounts of these metals are required by living organisms for their survival. The ranges of Pb and Ni in gill tissues of Asian swamp eel are 49.30 to 130.73 µg/g dw and 8.23 to 37.27 µg/g dw for 2011 and 2012, respectively. In comparison to previous study^30^, the Pb values were found to be in the range of 8.95 to 16.25 µg/g dw in gill tissues of six fish species gathered from the Mediterranean Sea. Besides, the high Pb and Ni levels might be extracted from agricultural activities that demand the utilization of excessive agrochemical fertilizers and pesticides in producing superior crop. Meanwhile, the minimum concentrations of Pb and Ni were found in the muscle of the eel for all the paddy seasons in 2011 and 2012. Furthermore, the Pb level in the muscle of the eel was discovered to be eight times higher than the permitted limits (2.00 µg/g) established by the Malaysian Food Regulation. This further proposes that the muscle of the Asian swamp eel is harmfully affected by Pb pollution.

Other parts of tissues, such as bone and skin, were also observed to moderately accumulate heavy metals (Zn, Cu, Cd, Pb, and Ni) for the paddy seasons in both 2011 and 2012. In this case, Cd metal was found to produce the minimum level of heavy metals. The removal of Asian swamp eel bone during food preparation may affect the real toxic level of heavy metals upon humans due to the reduced content of heavy metals. As for skin tissues, Zn concentration was found to be the highest, while the concentration of Cd was the lowest. It was reported that the presence of thick mucus layer on the skin surface acts as the barrier to safeguard the integrity of fish muscle tissues from being contaminated by surrounding pollutants^37^. Hence, this explains the higher Cd concentration in skin tissues, in comparison to muscle tissues. In most cases, muscle is utilized as one of the tissues to analyse heavy metals because it is widely consumed by most Kelantanese. In addition, most studies have employed fish muscle to assess its risks to human health^37^. However, in this study, most of the metals (Zn, Cu, Cd, Pb, and Ni) accumulated at the lowest concentrations in all the tissues selected for the purpose of this research (liver, gills, bone, skin, and muscle) for all the paddy seasons. These results mirror those of the past studies^16,22,39^.

### Estimation of potential health risk assessment

Fish and seafood are vital food sources for the general population of Malaysia because it is one of the cheapest protein sources. However, traces of harmful elements in fish and seafood may pose a terrible risk to human health. In addition, most individuals residing in Kelantan prefer consuming the muscle of Asian swamp eels due to its pleasant taste, soft texture, and high amount of protein. As such, the objective of this study is to evaluate the harmful effects of consuming the muscle of Asian swamp eels upon the general population of Malaysia. The regular consumption of trace elements had been predicted on the basis of the concentrations (wet weight basis) of trace elements in the muscle of Asian swamp eels, as well as the constant intake of the eels. According to the FAO^40^, the rate of fish consumption is 160 g/day for a Malaysian, 57 g/day for an Indonesian, and 85 g/day for a Thai. The average body weight of a 50 kg individual was used to investigate the annual fish consumption of an individual, which generated the result of 57.7 kg/year or 158 g/day in Malaysia^41^. The reference dose (RfD) for trace elements established by previous study are as follows: Zn (300 µg/kg body weight/day), Cu (40 µg/kg body weight/day), Cd (1 µg/kg body weight/day), Pb (3.57 µg/kg body weight/day), and Ni (20 µg/kg body weight/day) (Table 1).

In this study, the general intake of Pb metal accumulated in the muscle of Asian swamp eels for years 2011 and 2012 exceeds the established guideline value of 3.75 µg/day (Table 1). Fortunately, the regular amount of other metals, including Zn, Cu, Cd, and Ni, in the muscle of Asian swamp eel is lower than that of the established guidelines of fish consumption (Table 1). A possible explanation for this is that the muscle of this eel was affected by Pb pollution. Hence, a more discreet step should be taken to avoid Pb and Cd contaminations due to the regular intake of Cd metal of nearly 1 µg/day. This intriguing result leads to the possible harmful outcomes that might be caused by the consumption of paddy eel at the present rate.

The frequent symptoms caused by toxic heavy metals include fatigue and headache^30^. In fact, this study discovered that the high amount of Pb concentration in children and adults may lead to a wide range of health issues, such as convulsions, coma, renal failure, and fatality for the high-end to sub-lethal effects on metabolism, while intelligence was found to be affected due to low-end exposure^42^. In addition, toxicity of Cd can be the root of critical respiratory irritation, chronic lung disease, and testicular degeneration^43^. Therefore, knowledge on the appropriate level of heavy metal in fish is vital to be made known to the public, in order to ensure that both the nature and the human health are well-managed^30^.

### Limitation and recommendations

In this study, the sample size of the Asian swamp eels from the two paddy seasons was 163, while the eels were collected on the same area of paddy fields for two years paddy seasons in order to monitor the accumulation of heavy metals in the eels. However, to increase the accuracy of the results, it is suggested in the future to have bigger sample sizes for this kind of study, while the sampling period of two years should be extended.

However, as far as we are concern, due to the limitation of research related to Asian swamp eels in Malaysia, this present result is significantly important for current and future aspects in establishing the standard guidelines of heavy metals in fish in Kelantan, Malaysia. In addition, this available data could be used as guidelines for this country, and possibly the regional authorities, in planning sustainable agricultural activities without neglecting the impacts of pollution in environments.

## Conclusion

In conclusion, this study revealed that the concentrations of Cd and Pb are among the highest in the muscle of Asian swamp eels collected from the paddy cultivation areas located in Kelantan. Hence, this further implies that the regular intake of Cd and Pb metals from the muscle of the eels may exert several potential health-damaging effects, particularly heavy metals toxicity and cancerous problems. This situation gets worse when a majority of a population has been consuming the muscle of Asian swamp eels for a long period. On top of that, it is significant to establish the levels of heavy metals (Zn, Cu, Cd, Pb, and Ni) in varying tissues parts, such as liver, gills, bone, and skin of Asian swamp eel collected from different paddy seasons in Kelantan. Although, at current concentrations in Asian swamp eel had no metal was found to pose potential serious health risk, but collectively, the metals (Pb and Cd) are considered as potential human health hazards. Nevertheless, the intake of the muscle tissue of Asian swamp eels should be regulated consistently to avoid further critical health issues. The deposition of heavy metals through the excessive use of agrochemical fertilizers should be constantly observed and controlled by responsible government agencies.

## Materials and Methods

### Ethics statement

The eels were sampled, handled and sacrified according to methods approved by Institutional Animal Care and Use Committee, Universiti Putra Malaysia.

### Asian swamp eel collection and heavy metals analysis

**Figure 6:**
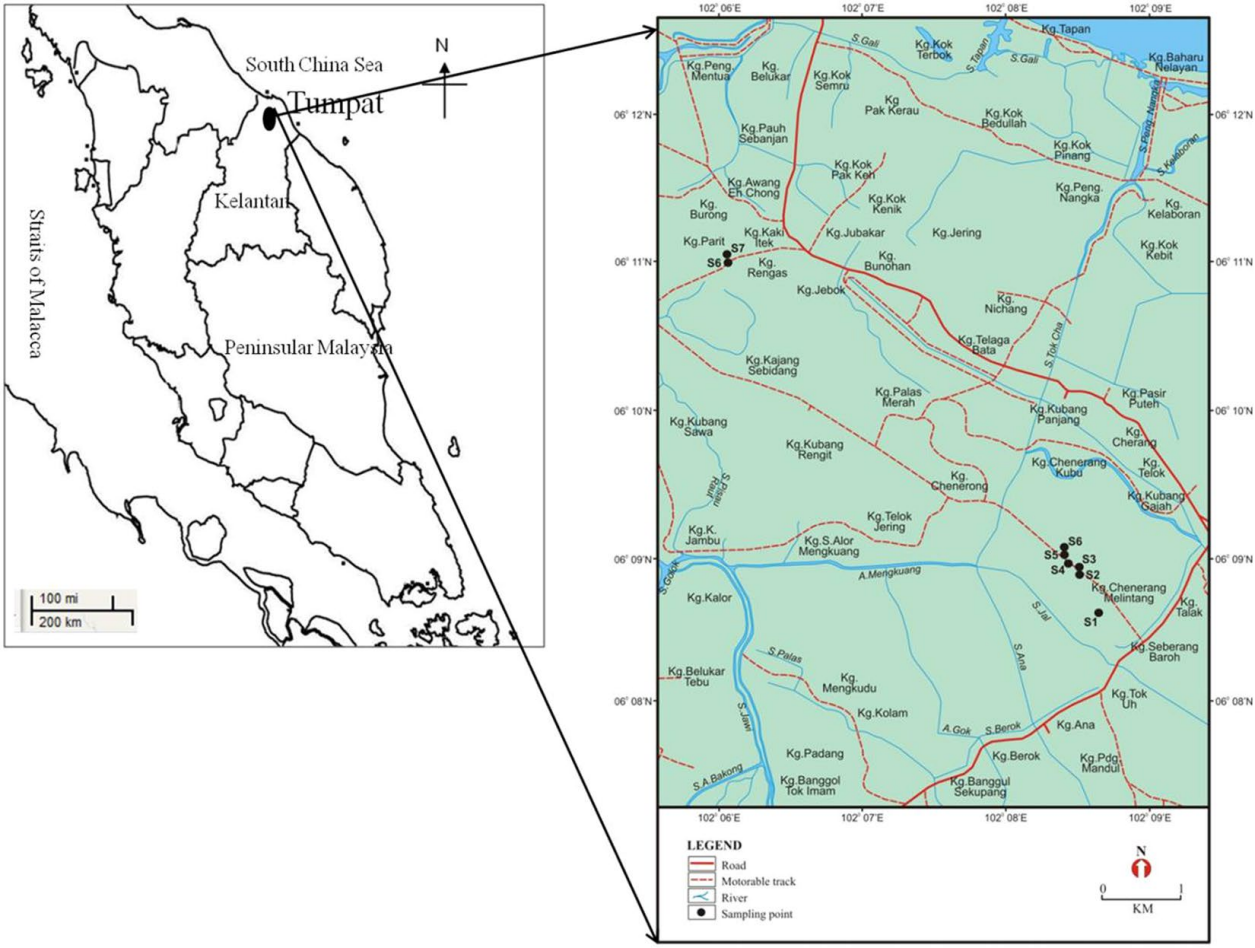
Sampling areas of Asian swamp eels situated in Kelantan in the year 2011 and 2012.

The Asian swamp eels gathered for this study had been obtained from a paddy field area located in Kelantan, Malaysia (Fig. 6). The study was conducted in two cycles, which was further categorized into four seasons, namely, ploughing, seedling, growing, and harvesting. The two phases were performed in varied years; 2011 and 2012. A locally developed tool, called “*tukil*”, was positioned upright and half-submerged in the paddy fields nearby crowded vegetation areas prior to the process of collecting data. The methods then followed by placing appropriate bait, such as cooked fish, to each *tukil* in order to allure the eel through it. All the *tukil* were then assembled in the following morning in order to assess the presence of the eel. The trapped paddy eels were placed into plastic bags and sent to the laboratory for further analyses^5^. The samples were acclimated for three days before dissection took place. The key guidelines that describe the total length and the total weight of the paddy eels were calculated by using the nearest centimetre (cm) and gram (g) unit, prior to the dissection procedure. It is also important to note that all the equipment utilized in this study had been cleaned to avoid contamination. A total of 163 eels were collected for the two-year paddy cycle. The obtained data showed that the average total length and body weight of the paddy eels were 46.41 cm and 106.97 g for the year 2011, while 43.72 cm and 92.75 g for the year 2012. Meanwhile, the concentration of heavy metals is expressed in the unit of µg/g dry weight.

The three parts of the eel tissues selected for the purpose of this research including liver, gill, skin, muscle, and bone. In general, bone and muscle were opted because they are widely consumed by a majority of the population in Kelantan. The sample tissues were dried in an air-circulating oven at 60 °C for 72 hours, until reaches a constant dry weight (dw). All the samples were then homogenised and 1.0 g of the samples was measured prior to acid digestion. Following that, the samples were digested using concentrated nitric acid (69%, AnalaR grade, BDH Chemicals, UK). Next, the digestion tubes were placed on the digestion block at 40 °C for 1 hour, which was then increased to 140 °C for 3 hours^44^. After a total of 4 hours of digestion, the samples were set aside for a 15 minutes in room temperature to let them suppress, while adding distilled water at a fixed volume of 40 mL. After that, the samples were refined using filtered paper (Whatman No. 1) and the filtrates were stored at 4 °C until the process of metal determination. As mentioned in the previous section, the heavy metals of Cu, Zn, Pb, Cd, and Ni were analysed by using the air-acetylene flame atomic absorption spectrophotometer (AAnalyst 880, Perkin-Elmer, USA). The data are presented in µg/g dry weight basis. Multiple-level calibration curves against which sample concentrations were calculated. Standard solutions were prepared from 1000 mg/l stock solution of each metal (MERCK Titrisol). All glassware used was first acid-washed via dipping for 16 hours, and next rinsed with distilled water for a period of time and air-dried prior to further use.

The DORM-3 Certified Reference Material (CRM) [National Research Council Canada (NRC 2007)] was employed for Zn and Cu metals for the purpose of verifying the accuracy of the adopted procedures. On another note, PACS-2 Certified Reference Material (CRM) [National Research Council Canada (NRC 2007) was employed for a number of metals, such as Cd, Ni, and Pb. The percentages of recoveries are as presented in Table 2; 91.12% for Zn, 109.17% for Cu, 126.82% for Cd, 83.21% for Ni, and 93.75% for Pb.

**Table 2:** Measured results (µg/g dry weight ± SD) of the Certified Reference Material for fish with certified values for Zn, Cu, Cd, Ni, and Pb.

Metal	Zn^a^	Cu^a^	Cd^b^	Ni^b^	Pb^b^
Measured	46.74 ± 4.74	16.92 ± 2.02	2.68 ± 0.33	32.87 ± 5.46	171.57 ± 15.48
Certified	51.3 ± 3.1	15.5 ± 0.6	2.1 ± 0.2	39.5 ± 2.3	183.0 ± 8.0
Recovery	91.12%	109.17%	126.82%	83.21%	93.75%

Remark: ^a^Certified Reference Material (DORM-3). ^b^Certified Reference Material (PACS-2).

### Estimation of chemical doses in tissues

The regular intake doses were calculated by using the established standard equation^45^, as shown below:

$$D=({C}_{{metal}}\ast {I}_{{food}{\rm{intake}}})/W$$

where,

$$\,\begin{array}{ll}D & {\rm{estimated}}\,{\rm{dose}}\,(\mu {\rm{g}}/\mathrm{kg}\,{\rm{body}}\,\mathrm{weight}/\mathrm{day})\,{\rm{for}}\,{\rm{chemical}}\,{i}\,{\rm{at}}\,{\rm{ingestion}}\,{\rm{rate}}\,{j}\,\\ {C}_{{metal}} & {\rm{concentration}}\,{\rm{of}}\,{\rm{chemical}}\,{i}\,({metal})\mathrm{on}\,{\rm{fish}}\\ {I}_{{food}{intake}} & {\rm{daily}}\,\mathrm{intake}/\mathrm{ingestion}\,{\rm{rate}}\,{\rm{of}}\,{\rm{fish}}\,{\rm{per}}\,{\rm{day}}\,{\rm{by}}\,{\rm{Malaysian}}\,{\rm{population}}\\ W & {\rm{assumed}}\,{\rm{human}}\,{\rm{body}}\,{\rm{weight}}\,(\mathrm{50}\,\mathrm{kg})\end{array}$$
